# Synthesis of an innovative SF/NZVI catalyst and investigation of its effectiveness on bio-oil production in liquefaction process alongside other parameters

**DOI:** 10.1007/s11356-024-32981-z

**Published:** 2024-03-25

**Authors:** Kübra Ersöz, Bahar Bayrak, Figen Gündüz, Hüseyin Karaca

**Affiliations:** 1 Engineering Faculty, Chemical Engineering Department, Atatürk University, 25240 Erzurum, Turkey; 2 Engineering Faculty, Chemical Engineering Department, Inonu University, Elazig Road 15Th Km, 44280-Campus, Malatya, Turkey

**Keywords:** Walnut shells, Silica fume, Nano zero-valent iron, Direct liquefaction, Total conversion, Oil yield

## Abstract

Today, new energy sources alternative to fossil fuels are needed to meet the increasing energy demand. It is becoming increasingly important to constitute new energy sources from waste biomass through the liquefaction process. In this study, walnut shells (WS) were liquefied catalytically and non-catalytically under different parameters using the liquefaction method. In this process, the effect of silica fume/nano zero-valent iron (SF/NZVI) catalysts on the conversion rates was investigated. The catalyst was synthesized by reducing NZVI using a liquid phase chemical reduction method on SF. The SF/NZVI catalyst was characterized by scanning electron microscopy- energy dispersive X-ray (SEM–EDX), transmission electron microscope (TEM), Brunauer–Emmett–Teller (BET), Fourier transform infrared spectroscopy (FTIR), X-ray diffraction (XRD), and X-ray photoelectron spectroscopy (XPS) analysis. The effect of various process parameters on the liquefaction process was investigated. In this context, the reaction temperature ranged from 300 to 400 °C, the solid/solvent ratio ranged from 1/1 to 1/3, the reaction time ranged from 30 to 90 min, and the catalyst concentration ranged from 1 to 6%. According to the results obtained, the most suitable operating conditions for non-catalytic experiments in liquefaction of WS were found to be temperature of 400 °C, reaction time of 60 min, and solid/solvent of 1/3. In catalytic conditions, the optimum values were obtained as temperature of 375 °C, reaction time of 60 min, solid/solvent ratio of 1/3, and catalyst concentration of 6%. The highest total conversion and (oil + gas) % conversion were 90.4% and 46.7% under non-catalytic conditions and 90.7% and 62.3% under catalytic conditions, respectively. Gas chromatography/mass spectrometry (GC/MS) analysis revealed the bio-oil was mainly composed of aromatic compounds (benzene, butyl-, indane and their derivatives,) and polyaromatic compounds (naphthalene, decahydro-, cis-, naphthalene, 1-methyl-.). The aim of increasing the quantity and quality of the light liquid product in the study has been achieved.

## Introduction

Fossil fuels have been meeting most of the world’s energy demand since ancient times. The world will imminently face the risk of resource depletion depending on the rate and amount of use of fossil fuels (Willauer and Hardy 2020). The direct burning of fossil fuels leads to an increase in the concentration of carbon dioxide in the atmosphere (Xue et al. 2011; Wu et al. 2018). According to a report by the International Energy Agency published in 2022, fossil fuels have been around 80% of the energy sources consumed globally for many years. In 2025, global energy-related CO_2_ emissions are expected to reach 37 billion tons (IEA 2022). The increase in CO_2_ emissions, which is one of the biggest causes of the increase in greenhouse gas emissions, leads to climate change (Sun et al. 2023). Also, it is projected that the global average temperature will increase by about 2.5 °C by 2100. Clean and renewable alternative fuels are being investigated due to the increase in energy consumption with the growing world population, the risk of resources depletion, and serious environmental problems caused by fossil fuels (Leng et al. 2020; Acharya et al. 2022).

Biomass energy is one of the most popular energy sources today, obtained by converting biomass into electricity, heat, power, or fuels (Wu et al. 2018; Kanca 2020; Benti et al. 2021). It is known that biomass resources provide approximately 10–14% of the world’s energy supply (Saxena et al. 2009). WS are a lignocellulosic solid containing approximately 35% lignin, 30% cellulose, and 25% hemicellulose in their structure (Almeida 2020). WS, as a biomass source, are the agricultural waste obtained from walnuts, which are widely cultivated in Central Asia, Turkey, Europe, Western China, and Iran (Shahbandeh 2023). Therefore, the quantity of WS as waste is quite high as walnut products are used in many areas such as food, medicine, paint, and furniture (Domingos et al. 2022). WS when burned or left as waste in the environment creates serious ecological problems. WS has great potential as a raw material for liquid fuel production.

Gasification, combustion, pyrolysis, enzymatic hydrolysis pathways, biodiesel, and fermentation processes are used to obtain clean fuel (Bedir and Doğan 2021; Osman et al. 2021). This method aims to obtain clean fuel, synthesis gases, and chemical raw materials by increasing the solubility of the raw material in a suitable solvent and removing heteroatoms. During liquefaction, macromolecular compounds in the structure of the raw material are broken down into light molecules under catalytic or non-catalytic conditions. The unstable and reactive molecules formed after fragmentation are re-polymerized into lighter compounds by saturation with a hydrogen-donating solvent (Demirbaş 2000; Mochida et al. 2014). For many years, studies have been carried out using many different raw materials in fuel production by direct liquefaction method (Shabbirahmed et al. 2022). Chang et al. achieved a conversion ratio of approximately 92% at 360 °C in their liquefaction study with Wucaiwan coal (Chang et al. 2019). In another study, tetralin was used as a hydrogen donor, resulting in 75% (oil + gas), 5.1% asphaltene, and 4.8% pre-asphaltene as liquefaction products in 85% total conversion (Ali et al. 2014). A wide variety of waste materials such as pine wood, sunflower stalks, sawdust, polypropylene, and pet have been used in the production of liquid fuel by liquefaction (De Caprariis et al. 2017; Isa et al. 2018; Olam and Karaca 2023). Wang et al. found that the oil yield ranged between 6.8 and 67.1% in the liquefaction of sawdust with tetralin at a temperature of 200–350 °C, a pressure of 4–10 MPa, and a reaction time of 10–100 min (Wang et al. 2007). In another study, an oil yield of approximately 19–35% was obtained in experiments carried out with sodium carbonate catalyst and carbon monoxide gas from moss, water hyacinth, waste grain, sorghum field residue, and Napier grass (Elliott et al. 1988).

The use of catalysts to increase total conversion and oil yield in liquefaction is quite common. In a study, various catalysts were used to investigate the degradation of diphenyl ether at 350 °C under catalytic conditions. It has been determined that the most effective catalyst for total conversion is Fe_2_O_3_ (Matsuhashi et al. 1997). In another liquefaction study, an approximately 87% total conversion was achieved using a catalyst synthesized by adding cerium (Ce) to cellulose by the precipitation method (Liu et al. 2019). Nanocatalysts have recently been used in different industries, including fuel production from biomass (Li et al. 2008; Akia et al. 2014). The effect of a nanocatalyst (nano-Ni/SiO_2_), an acid catalyst (zeolites), and an alkali catalyst (Na_2_CO_3_) was investigated to increase the oil yield at low temperatures in the liquefaction of algae and the highest yield was found in the presence of nanocatalyst (Saber et al. 2016). Among these catalysts, nanosized iron-based materials are common due to their superior catalytic activity and low cost. In a study conducted with the Fe_3_O_4_ nanocatalyst, the total conversion and oil yield of coal were obtained as 96.6% and 60.4%, respectively, under optimal reaction conditions (Li et al. 2017). The total conversion, oil yield, and liquefaction degree of local coal with another iron-based nanocatalyst coated with oleic acid were found to be 97.2%, 86.5%, and 92.0%, respectively. In another study, the percentages of total conversion, oil yield, and liquefaction degree with oleic acid coated β-FeOOH were found to be 92.6%, 73.8%, and 80.8%, respectively. It will be possible to increase the efficiency of industrial direct liquefaction processes in the future with these nanocatalysts (Li et al. 2012; Sheng et al. 2017).

When catalysts are used in the nanoscale, they provide high catalytic activity for reactions due to the increase in surface areas and the number of active centers on the surface. Fe, Cu, Ni, Pd, Au, and Ag are the most used nanocatalysts (Ye et al. 2022). Especially iron nanoparticles play an active role in the formation and breakdown of carbon–carbon bonds (Yılmaz 2011). However, nanoscale zero-valent iron alone is difficult to recycle and reuse because it is very small, easily agglomerates, and undergoes undesirable reactions (Yıldırım and Bayrak 2022). In order to eliminate these disadvantages, NZVI is distributed homogeneously on support material (activated carbon, metal oxide, zeolite, etc.) with a high surface area (Gündüz and Bayrak 2018). Components such as natural clays, alumina, zinc oxide, silica, and activated carbon are generally used as support in catalyst production (Hoşgün 2018). In recent years, many studies have been carried out with supporting material/NZVI composite (Xiao et al. 2015; Yang et al. 2016; Chen et al. 2023, 2024; Leovac Maćerak et al. 2023). SF, which is the support material in our study, is typically discarded as waste in silicon metal production. SF possesses a large surface area, making it an excellent support material. For this reason, it is expected that the synthesized SF/NZVI composite will serve as an effective catalyst in the biomass liquefaction process.

In this study, a new SF/NZVI catalyst was synthesized to increase both the amount and quality of the light product obtained during the liquefaction of WS. At the same time, optimum process parameters were determined under catalytic and non-catalytic conditions. Since the primary aim of liquefaction is to increase the (H/C)_atomic_ ratio, the synergistic effect on liquefaction products was investigated by increasing the (H/C) ratio with the catalyst used in this study. The main objective of this study is to produce alternative fuels to petroleum by direct liquefaction of WS and to valorize harmful environmental waste.

## Materials and methods

### Material

WS used in liquefaction experiments was obtained from Malatya Doğanşehir, Turkey. Waste SF used as support material in the preparation of SF/NZVI catalyst was obtained from Antalya Eti Electrometallurgy Corporation.

### Preparation of SF/NZVI catalyst

The supplied SF was sieved to a size of < 0.075 mm and stored in closed containers. The synthesis of the SF/NZVI catalyst was carried out using the liquid phase reduction method (Yıldırım and Bayrak 2022).

$$2\text{Fe}^{2+}+{2\text{BH}}_{4}^-+{6\text{H}}_2\text{O}\rightarrow\text{Fe}^0\downarrow+2\text{B}{\left(\text{OH}\right)}_3+{7\text{H}}_2$$

Firstly, 1.83 g of Iron II chloride tetrahydrate (FeCl_2_.4H_2_O) (99%, Acros Organics) was dissolved 30% (v/v) in 50 ml of ethanol. Then 0.5 g of polyethylene glycol (PEG) (Merck KGaA, Germany) as the surfactant and 1 g of SF were added. This solution was mixed under nitrogen in a 3-necked flask for 1 h. After 100 ml of 1.61 mol/L sodium borohydride (NaBH_4_) (Merck KGaA, Germany) solution was prepared with ethanol (99.9%, SigmaAldrich, St. Louis, MO), it was added to the flask at a rate of 30 drops/min.

Finally, the mixture was stirred with nitrogen gas for another 30 min, and the solution was separated from the solid by centrifuging. The separated solid particles were washed with 30% ethanol, dried at 40 ^0^C for 5 h, and stored in closed containers.

### Characterization of SF/NZVI catalyst

Characterization of SF and SF/NZVI catalyst was performed at Atatürk University Eastern Anatolia High Technology Application and Research Center (DAYTAM). Their surface images were taken using Zeiss/ Sigma 300 brand SEM–EDX and Hitachi/HT 7700 brand TEM. The surface area of SF/NZVI was determined using a Micromeritics/3 Flex 4.02 BET surface analyzer, with specific surface areas determined at 77 K under liquid nitrogen. The FTIR spectra of SF and SF/NZVI were determined with a Bruker VERTEX 70v model FTIR device at a wavelength range of 400–4000 cm^−1^. XRD patterns for the SF, SF/NZVI catalyst, and NZVI were performed using a GNR Explorer model XRD at 2θ = 10°–100° with a scan rate of 1°/min. The chemical structures on the surface of the SF/NZVI catalyst were investigated using both general and partial scanning techniques with a Specs-Flex model XPS instrument.

### Direct liquefaction experiments

Liquefaction experiments were carried out under catalytic and non-catalytic conditions. The effect of reaction temperature, reaction time, solid/solvent ratio, and catalyst concentration parameters on liquefaction performance was examined. The liquefaction conditions were given in Table 1.

**Table 1:** Liquefaction conditions

Exp. No	Temperature (°C)	Time (min)	Solid/solvent ratio	Catalyst concentration (wt %)	Catalyst type
1	300	60	1/3	-	-
2	325	60	1/3	-	-
3	350	60	1/3	-	-
4	375	60	1/3	-	-
5	400	60	1/3	-	-
6	375	30	1/3	-	-
7	375	90	1/3	-	-
8	375	60	1/1	-	-
9	375	60	1/2	-	-
10	375	60	1/3	1	SF/NZVI
11	375	60	1/3	3	SF/NZVI
12	375	60	1/3	6	SF/NZVI

**Fig. 1:**
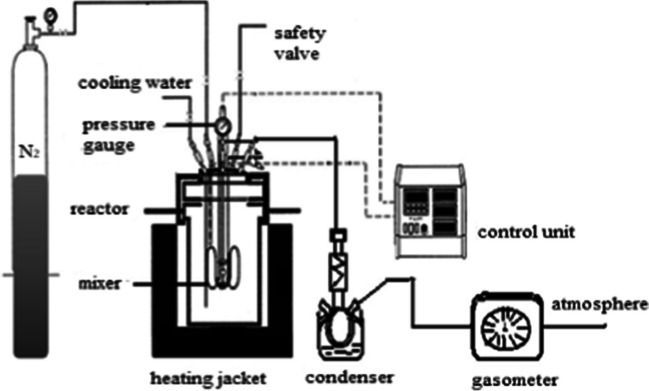
Pressurized batch reactor system

WS was used as biomass in liquefaction experiments. Initially, the walnuts were broken and brought to the size of 0.2–0.25 mm after their fruits were separated. WS was loaded into a 500-ml autoclave (PARR 4575/4842) with tetralin solvent (99%, Merck KGaA, Germany) under a nitrogen atmosphere (Fig. 1). Nitrogen gas was introduced into the system to purge air from the batch reactor and then the gas outlet valves were closed and the reactor was pressurized to 20 bar with N_2_ gas. The reactor was heated to the specified temperature and the temperature was maintained constant throughout the reaction time. After completion of the reaction, the autoclave was cooled and the amount of gas inside was measured.

**Fig. 2:**
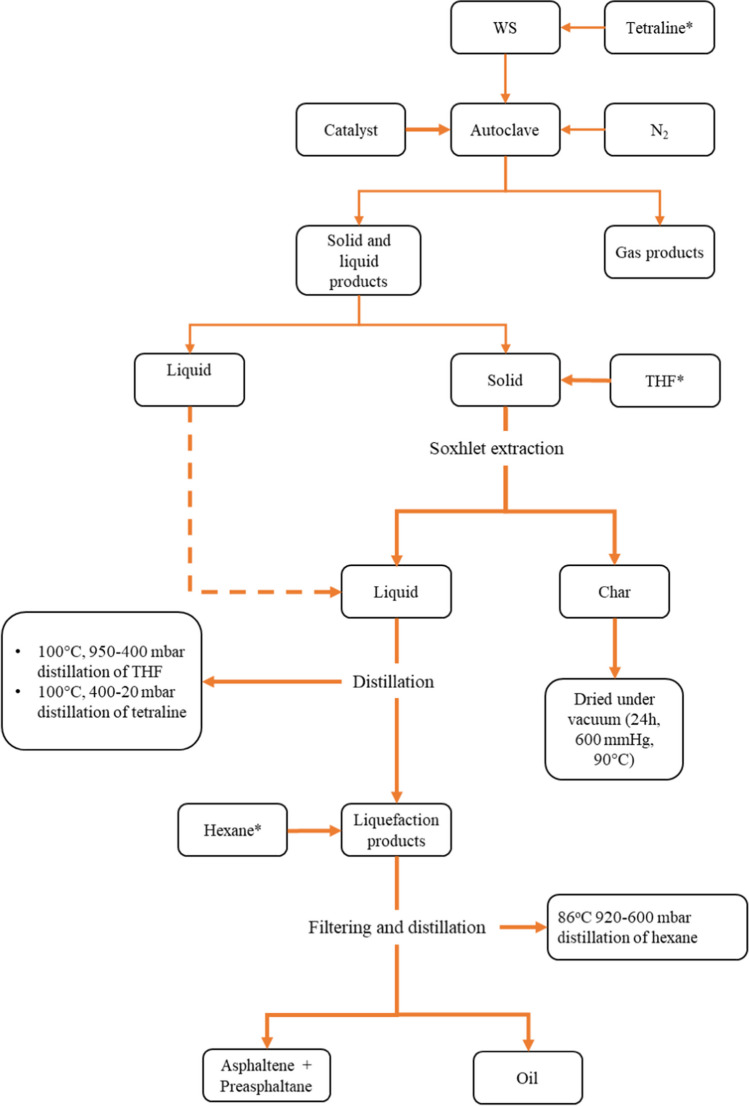
Experimental flow chart for liquefaction process

After the reaction, the solid and liquid products in the reactor were separated by filtration. Residues remaining in the reactor were taken by washing with 250 ml of Tetrahydrofuran (THF) (99.9%, Sigma-Aldrich, St. Louis, MO). The liquid products remaining in the solid product were extracted with THF using a Soxhlet apparatus. The tetralin and THF used were recovered under vacuum by a rotary evaporator (Buchi R-200 Rotavapor). The liquid products collected from Soxhlet extraction and filtration were mixed with 250 ml of hexane (99%, Sigma-Aldrich, St. Louis, MO) in a flask using a magnetic stirrer (Weightlab WN-H550) for 15 h, and then this mixture was filtered. The filtrate contained hexane and oil products. The dissolved fractions were gradually separated from the hexane in an evaporator under a vacuum of 920 mbar, allowing for the recovery of hexane and separation of the oil product. The fraction remaining on the filter paper contained the Pre-asfalten (PAS) and Asfalten (AS) being heavy liquefaction products and was obtained by drying at 100 °C for about 1 day. The THF-free solid product obtained after the reaction was kept in a vacuum oven at 600 mmHg pressure and 90 °C temperature until completely dry and stored in closed containers (Fig. 2).

### Analyses

The products obtained after liquefaction were calculated by the equations given below.

1 $$\mathrm{Char\;yield\%}=\frac{\mathrm{char\;amount}({\text{g}})}{{\text{sample}}({\text{g}})}.100$$

Total conversion rate (Liquefaction products + gases);

2 $$\mathrm{Total\;conversion\%}=100-\mathrm{char\;yield\%}$$

Calculation of yields of liquefaction products:

PAS yield;

3 $$\mathrm{PAS\%}=\frac{{\text{PAS}}({\text{g}})}{{\text{sample}}({\text{g}})}.100$$

AS yield;

4 $$\mathrm{AS\%}=\frac{{\text{AS}}({\text{g}})}{{\text{sample}}({\text{g}})}.100$$

Oil + gas yield;

5 $$\left({\text{Oil}}+{\text{Gas}}\right)\mathrm{\%}=\mathrm{Total\;conversion\%}-(\mathrm{PAS\%}+\mathrm{AS\%})$$

Qualitative and quantitative analyses of the oils obtained from biomass liquefaction under catalytic and non-catalytic conditions were carried out at Atatürk University Eastern Anatolia High Technology Application and Research Center (DAYTAM). The analyses were performed using Shimadzu brand GC–MS QP2010 Ultra instrument. The DB-1701 column used for the analysis has a length of 15 m and a diameter of 0.25 mm, with a film thickness of 0.25 μm. Helium was used as the carrier gas at a flow rate of 1.5 cm^3^/min. Samples were dissolved in hexane and injected into the instrument at an injection volume of 0.1 μL. Analysis conditions were given in Table 2.

**Table 2:** Analysis conditions of the GC–MS device

Detector temperature	240 °C	Injector temperature	230 °C
Oven temperature program			
Starting temperature	60 °C	Heating rate	20 °C/min
Final temperature:	235 °C		

Proximate analyses for biomass and chars were performed according to ISO 1171:2010 and ASTM D-3172–07 standards (ISO 2010; ASTM 2013). The calorific values of chars obtained from liquefaction and WS were determined using the IKA C200 brand calorimeter. Elemental analyses were carried out at İnönü University Scientific and Technical Research Center (İBTAM) using a LECO brand CHNS-932 device. When the sum percentage of the carbon (C), hydrogen (H), nitrogen (N), and sulfur (S) percentages obtained from the elemental analyzer was subtracted from 100, the percentage of oxygen was found.

6 $$O\%=100-(C\%+H\%+N\%+S\%)$$

## Results and discussion

### Characterization of SF/NZVI catalyst

**Fig. 3:**
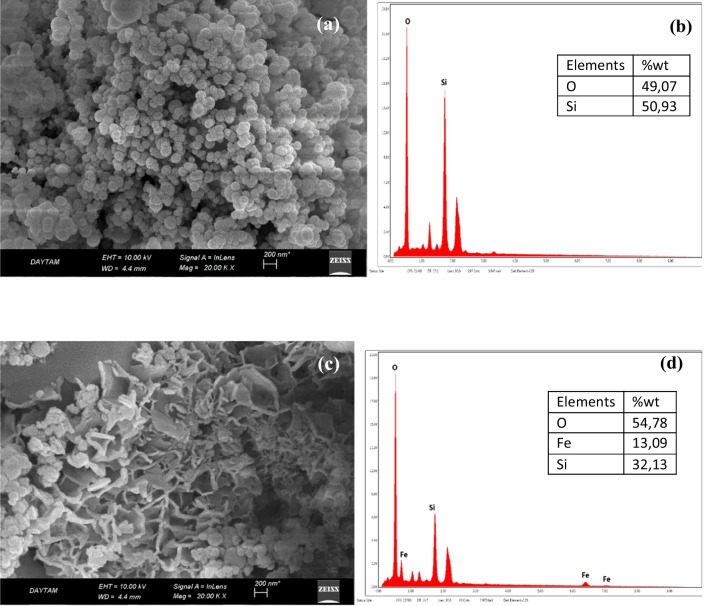
**a** SEM image of SF, **b** EDX analysis of SF, **c** SEM image of SF/NZVI, and **d** EDX analysis of SF/NZVI

SEM images of SF and SF/NZVI were given in Fig. 3. In the SEM image of the SF shown in Fig. 3a, it was seen that it had a small particle structure and the surface was smooth. Since SF contained SiO_2_ of more than 91%, Si and O peaks were predominantly visible in the EDX analysis (Fig. 3b). When the SEM/EDX analysis for SF was examined in Fig. 3b, peaks were observed at 1.74 keV for silicon and 0.52 keV for oxygen. In the SEM images of SF/NZVI, it was observed that new nano flake structures were formed on the surface (Fig. 3c). Figure 3d showed the EDX analysis for SF/NZVI. It was observed that there were iron peaks reduced on the surface in Fig. 3d spectra when compared to the spectrum of Fig. 3b (Gündüz and Bayrak 2018). In Fig. 3d, peaks belonging to iron were obtained at 0.7, 6.4, and 6.99 keV.

**Fig. 4:**
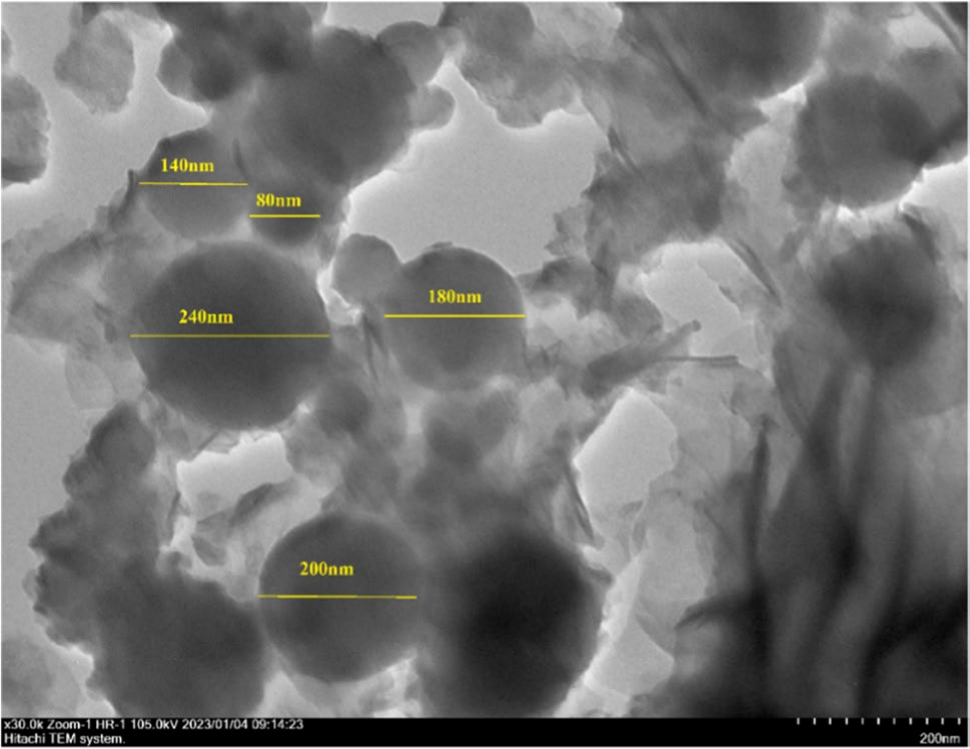
TEM micrograph of SF/NVZI

The TEM micrograph of SF/NZVI was given in Fig. 4. Iron nanoparticles reduced by the liquid phase chemical reduction method on the SF support material were found to be approximately 80–240 nm in diameter (Yıldırım and Bayrak 2022). Iron nanoparticles on the SF were homogeneously dispersed on the surface and well-separated. Differences in the size of nanoparticles have occurred as a result of their aggregation due to reasons such as the magnetic properties of particles, and the tendency to thermodynamically favorable state (Wang et al. 2014).

BET surface area of SF was measured as 22.82 m^2^/g, while it was determined to be 44.13 m^2^/g for SF/NZVI modified by bounding nano-iron. The surface area of SF/NZVI was doubled because of the newly formed nanoflake structures with the modification as seen in the SEM images (Gündüz and Bayrak 2018).

**Fig. 5:**
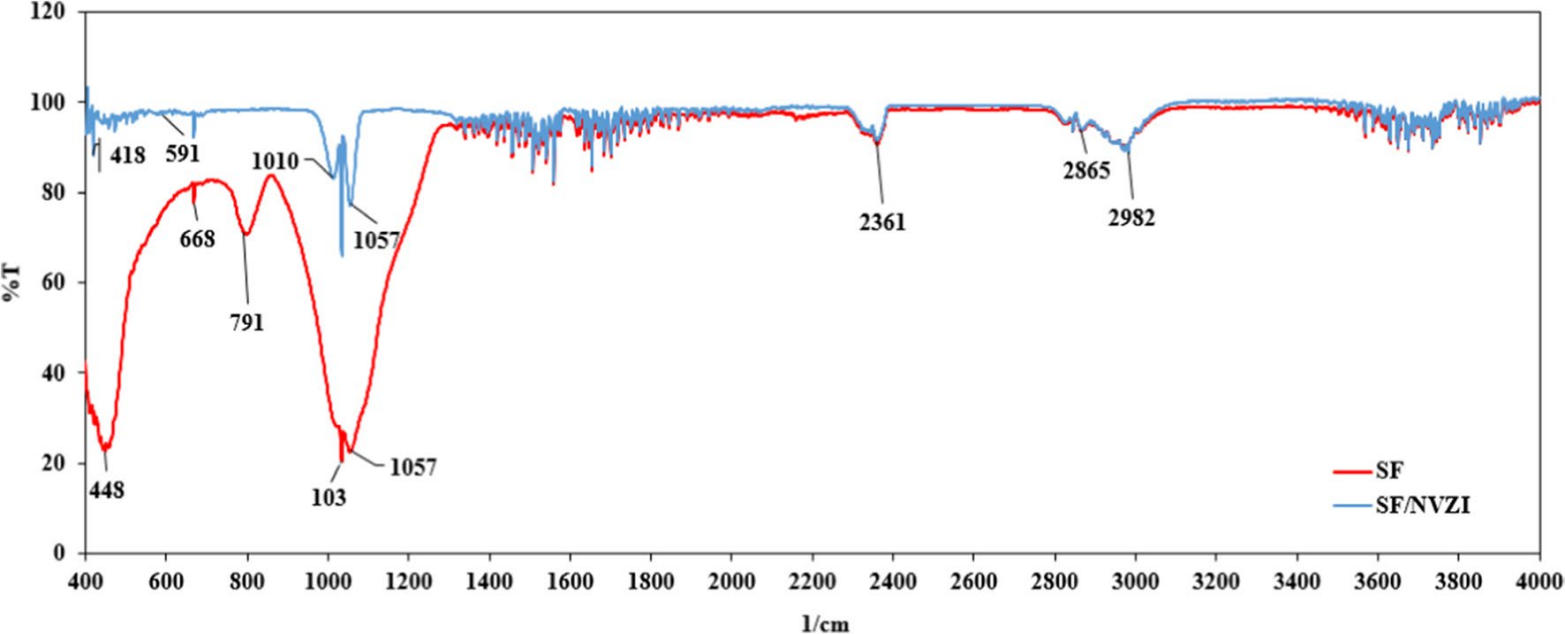
FTIR spectra of SF and SF/NZVI

The FTIR spectra of SF and SF/NZVI were given in Fig. 5. Due to the high SiO_2_ content in SF, the FTIR spectrum exhibited numerous bands corresponding to Si–O bonds. 448 cm^−1^ band in the spectrum of SF indicated O-Si–O bending vibration in Fig. 5 (Petreanu et al. 2023). Similarly, 1034 cm^−1^ and 1057 cm^−1^ peaks in both SF and SF/NZVI spectrums represented Si–O-Si and Si–O asymmetric stretching, respectively (Stuart 2004). Additionally, 791 cm^−1^ band observed in the SF spectrum gave Si–OH symmetric stretching vibration (Petreanu et al. 2023). The 2361 cm^−1^ band indicated the stretching of Si–H in large atoms such as silicon present in SF (Stuart 2004).

Iron oxides were formed by the oxidation of nano-zero valent iron bonded on SF, and the 1010 cm^−1^ band on the SF/NZVI spectrum indicated the M = O stretching formed between the metal and oxygen (Stuart 2004). The 668 cm^−1^ band was due to the vibration of Fe–O bonds on NZVI structures (Le et al. 2022). While the 590 cm^−1^ band gave the stretching vibration of the Fe–O bond (Zhao et al. 2014), the band observed at 418 cm^−1^ was thought to belong to hematite (Fe_2_O_3_) (Ali et al. 2018; Jiang et al. 2019).

**Fig. 6:**
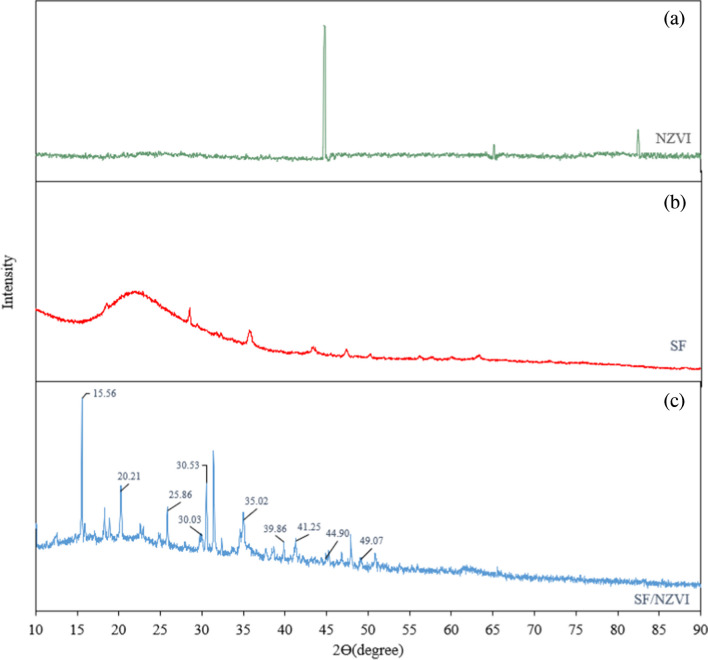
The XRD patterns of NZVI (**a**), SF (**b**), and SF/NZVI (**c**)

The XRD patterns for the SF, SF/NZVI catalyst, and NZVI were shown in Fig. 6. The peaks indicating the presence of Fe^0^ were observed at 44.71°, 65.08°, and 82.42° in the XRD pattern of NZVI (Fig. 6(a)). When Fig. 6(c) was examined, a peak showing the presence of Fe^0^ was seen at 44.71°. However, the intensity of the peak was low, since the amount of Fe bound on it, was less than the amount of supporting material (Wu et al. 2013).

While SF/NZVI was produced, some of the Fe^o^ could convert to magnetite (Fe_3_O_4_), maghemite (Fe_2_O_3_), and goethite [FeO(OH)]. Peaks observed at 2θ = 30.03°, 30.44°, 30.53°, and 35.02° in Fig. 6c may belong to the Fe_2_O_3_ or Fe_3_O_4_ peaks (Eljamal et al. 2018; Seyedi et al. 2022). Similarly, peaks at 2θ = 15.56°, 39.82°, 41.35°, 49,15° could be attributed to at FeO(OH) (Yang et al. 2016; Gündüz and Bayrak 2018). The SF forming the supporting material contains a high percentage of SiO_2_. Therefore, peaks at 20.21° and 25.83° may correspond to SiO_2_ identified as quartz in Fig. 6b (Liu et al. 2012).

**Fig. 7:**
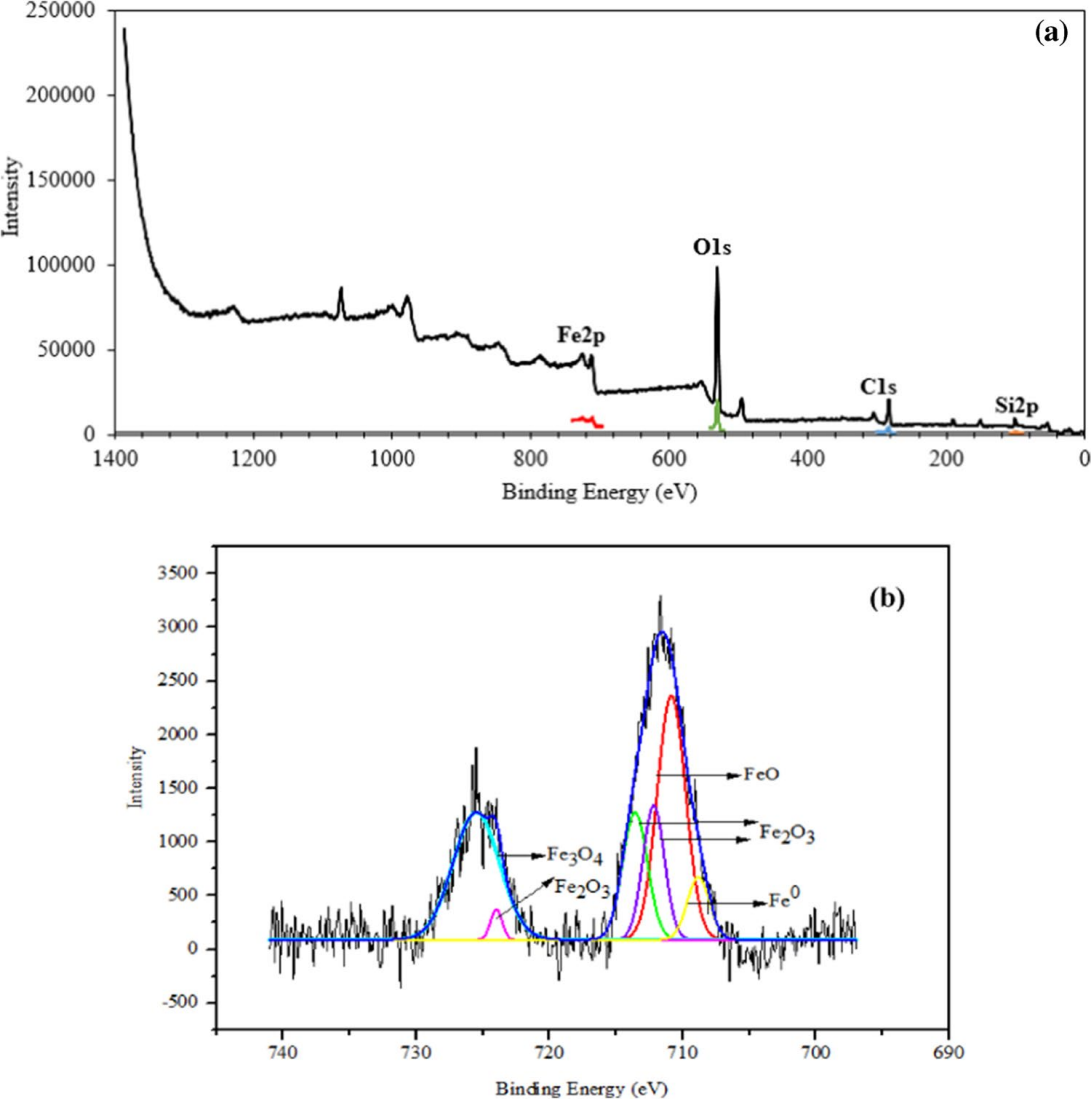
XPS survey spectra of SF/NZVI (**a**) and Fe2p spectra of SF/NZVI (**b**)

The XPS spectrum for SF/NZVI is provided in Fig. 7. Figure 7(a) presented the survey spectra while Fig. 7(b) showed the spectrum corresponding to the Fe 2p region. The Fe 2p_3/2_ peak was found to be larger compared to Fe 2p_1/2_. The peak position for Fe 2p_3/2_ was determined to be 711.6 eV, while for Fe 2p_1/2_, it was found to be 725.3 eV (Yamashita and Hayes 2008). Fe^0^ exhibited peaks at 707 eV in the Fe2p region. When iron oxidized, FeO was obtained at 710.7 eV, Fe_2_O_3_ gave peaks at 711.6, 712.2, and 724.1 eV, and Fe_3_O_4_ showed a peak at 725.8 eV (Yi et al. 2019). The deconvolution results showed that the oxidized iron particles in the SF/NZVI sample contained 31.07% Fe_2_O_3_, 33.57% FeO, 28.64% Fe_3_O_4_, and 6.72% Fe^0^.

### Analysis of raw samples

The results of proximate, elemental, and calorific value analyses of WS used in liquefaction were given in Table 3. The moisture content should be less than 10%, and the volatile matter content should be more than 65% to be used in the liquefaction of biomass sources. According to the analysis results, the selected WS was a suitable source in terms of % moisture and % volatile matter composition. The high amount of ash increases the amount of biochar obtained in liquefaction (Büyük 2017). In order to obtain high-quality bio-oil, the ash % must be low as in WS (Batur 2017). The H/C ratio of the biomass to be used was determined based on elemental analysis results. It was thought that the high (H/C)_atomic_ value was effective in facilitating hydrogen transfer to the free radicals formed on the biomass in the liquefaction process, thus increasing liquefaction efficiency (Coşkun 2020). The (H/C)_atomic_ ratio is expected to be around 1.4 in biomass with a lignocellulose structure (Varol 2015). The H/C ratio of WS was determined to be 1.44.

**Table 3:** Proximate, elemental, and calorific value analysis results of WS

Proximate analysis (wt % as used)	
Moisture	8.99
Ash	1.89
Volatile matter	80.21
Fixed carbon*	8.91
Elemental analysis(wt % daf)	
C	48.75
H	5.85
N	0.46
S	0.04
O*	44.90
(H/C)_atomic_	1.44
Higher heating value(kcal/kg)	3646

^*^Calculated from the difference, *daf*, dry ash-free

### Variation of temperature and pressure with time during the liquefaction process

**Fig. 8:**
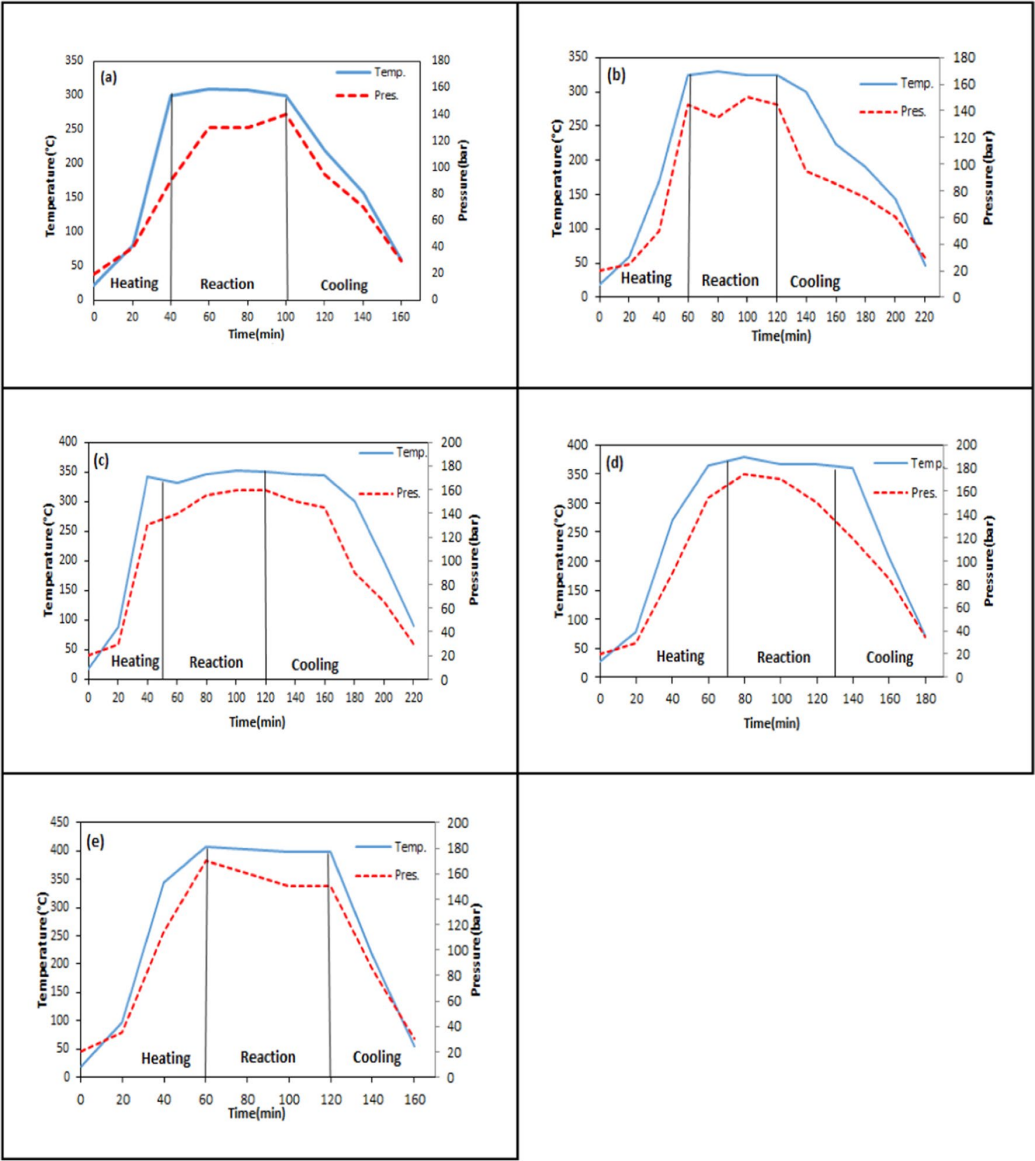
Temperature–pressure variation for different reaction temperatures at 60 min and 1/3 solid/solvent ratio (**a** 300 °C, **b** 325 °C, **c** 350 °C, **d** 375 °C, **e** 400 °C)

**Fig. 9:**
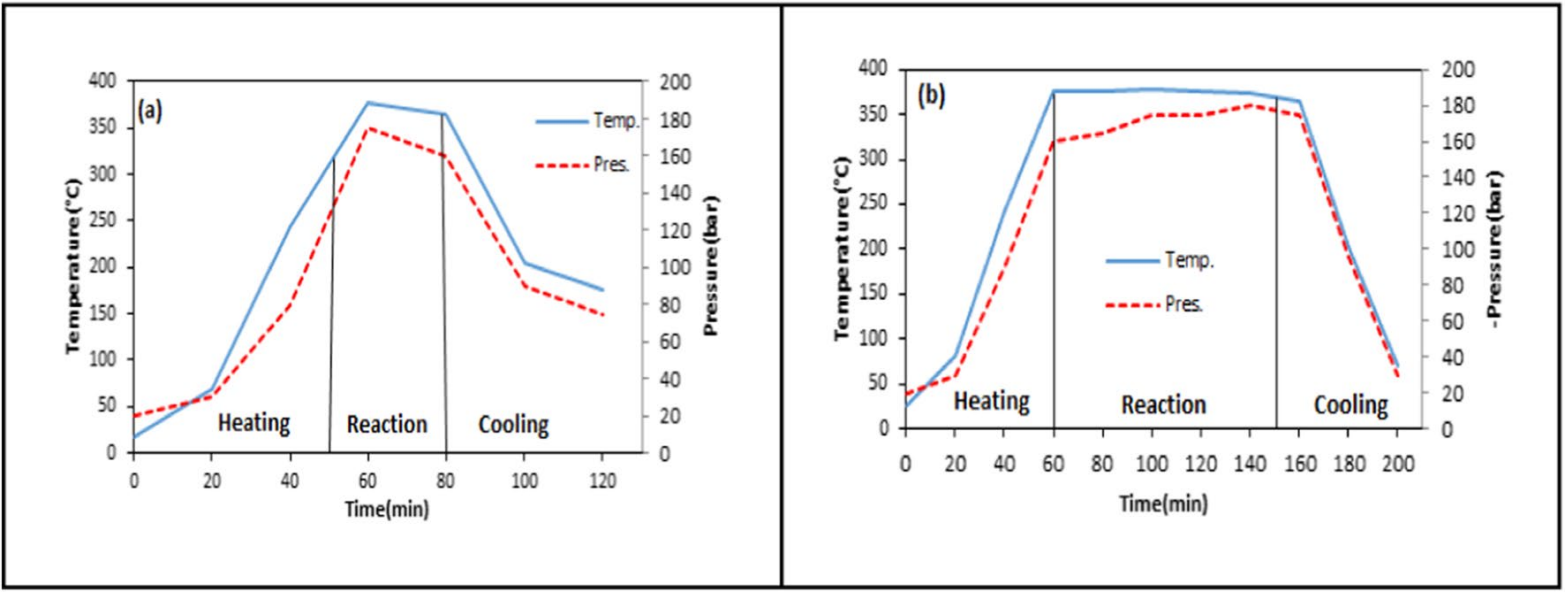
Temperature–pressure variation for different reaction times at 375 °C and 1/3 solid/solvent ratio (**a** 30 min, **b** 90 min)

**Fig. 10:**
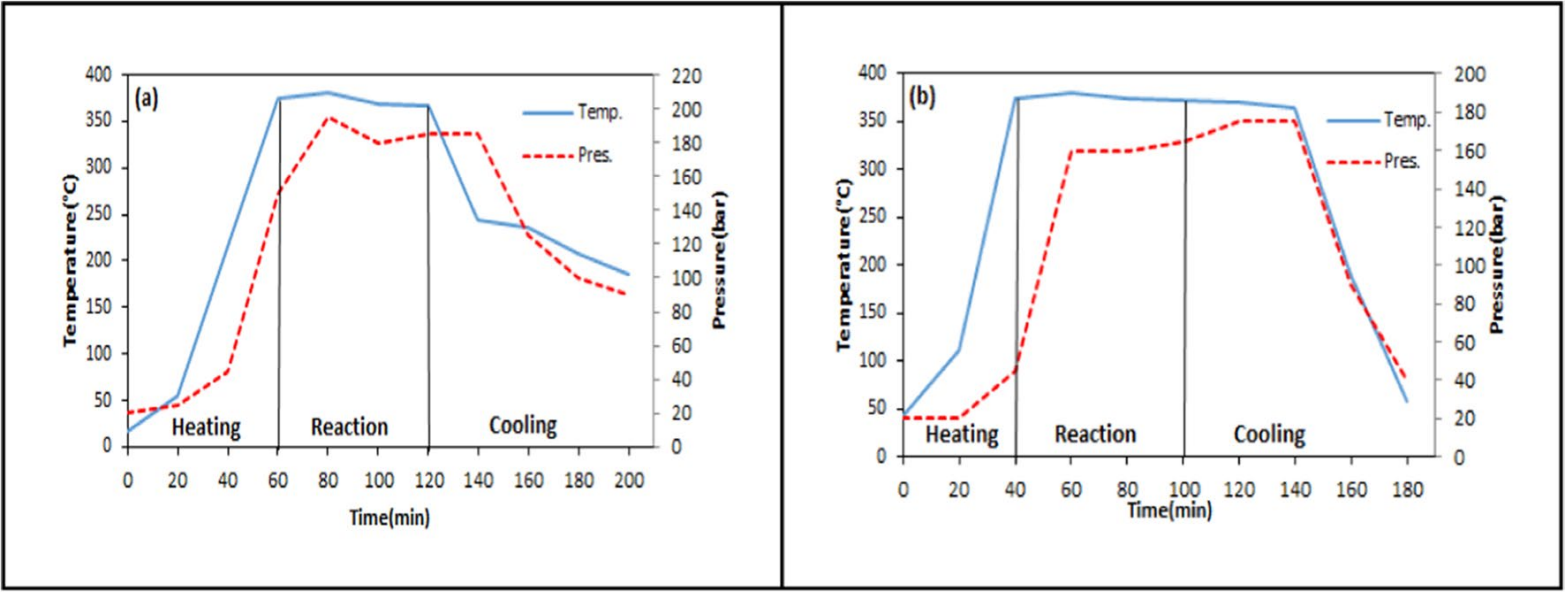
Temperature–pressure variation for different solid/solvent ratios at 375 °C and 60 min (**a** 1/1, **b** 1/2)

**Fig. 11:**
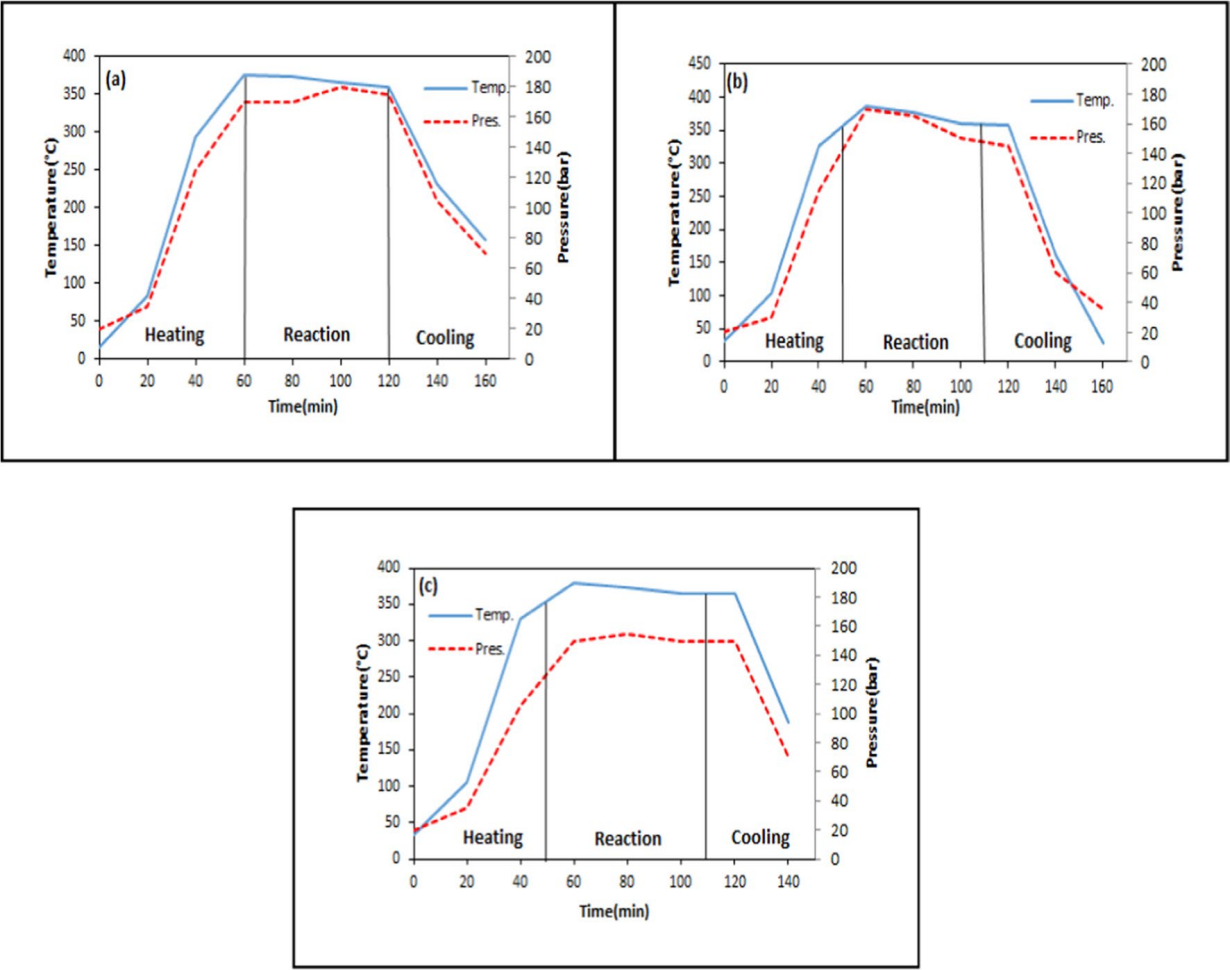
Temperature–pressure variation for different catalyst concentrations at 375 °C, 60 min, and 1/3 solid/solvent ratio (**a** 1%, **b** 3%, **c** 6%)

The temperature and pressure changes depending on time in direct liquefaction of WS were given in Figs. 8, 9, 10, and 11. It was found that the average heating rate was 5 °C/min. The desired temperature in the liquefaction experiments was reached in approximately 60 min, but this duration may slightly vary depending on process variables. After the system reached to the desired reaction temperature, the heating was maintained for a certain reaction time, and during which no significant changes in temperature were observed. This showed that the liquefaction experiments were carried out sensitively. After the reaction time was over, the system was cooled using a refrigerant.

In Fig. 8, temperature and pressure changes were examined for the liquefaction experiments performed at five different reaction temperatures (300–325-350–375-400 °C). The reaction temperature plays a crucial role in efficiently breaking down the biomass used in liquefaction and consequently increasing the yield of liquid product. As seen in Fig. 8, while the value of pressure was 140 bar at 300 °C, it reached 175 bar at 400 °C. The increase in pressure depending on the temperature showed that the formation of (liquid + gas) product increased [52].

In Fig. 9 and Fig. 8d, the pressure and temperature changes for liquefaction experiments performed at three different reaction times (30–60-90 min) were seen. The duration of the reaction time is critical to ensure sufficient conversion of biomass into free radicals and adequately contacted with the solvent. According to the results, while the pressure reached 175 bar in 30 min, it increased to a maximum of 180 bar in 90 min. No significant change was observed between the pressure readings at 30 and 90 min (Ersöz 2023).

In Fig. 10 and Fig. 8(d), temperature and pressure changes were investigated in liquefaction experiments with three different solid/solvent ratios (1/1–1/2–1/3). As the quantity of solvent increased, more hydrogen was introduced into the reaction medium. Thus, the homogenization increased as the free radicals formed were saturated with more hydrogen. While a maximum pressure of 200 bar was obtained in 1/1 solid/solvent ratio, 175 bar pressure was obtained in 1/3. No significant change in pressure was observed in this parameter (Chang et al. 2019, Ersöz 2023).

In Fig. 11, temperature and pressure changes were investigated in experiments performed at three different catalyst concentrations (1%-3%-6%). In Fig. 8(d), the temperature–pressure variation is shown for the non-catalytic test under the same conditions. In catalytic liquefaction experiments, hydrogen transfer to free radicals occurs mostly through the catalyst. The pressure remained relatively stable around 170 bar for all catalyst concentrations.

### Liquefaction experiments results

The char yields and total conversions obtained from liquefaction experiments performed under the influence of parameters such as temperature, solid/solvent ratio, reaction time, and catalyst concentration were given in Table 4. The total conversion increased from 80.9% at 300 °C to 90.4% at 400 °C in experiments carried out at different temperatures. When the temperature is more than 375 °C, the total conversion increases as the biomass is better broken down (Koyunoğlu 2010). Since the solvent (tetralin) degrades at temperatures above 425 °C, it cannot be possible to work at temperatures higher than this temperature (Çavuşoğlu et al. 2022). The highest conversion rate was found as 90.4% at 400 °C, 1/3 solid/solvent ratio, 60 min for non-catalytic conditions and 90.7% at 375 °C, 1/3 solid/solvent ratio, 60 min, and 6% catalyst concentration for catalytic conditions. The reaction time should be long to ensure sufficient contact with the solvent after the breakdown of the biomass. When the effect of reaction time was analyzed, it was found that the total conversion was seen to change from 89.7% at 30 min to 90.2% at 90 min (Ersöz 2023).

**Table 4:** Liquefaction experiment results

Exp. No	Temperature (°C)	Solid/solvent	Time (min)	Catalyst concentration (wt %)	Char yield (daf, %)	Total conversion (daf, %)
1	300	1/3	60	0.00	19.08	80.9
2	325	1/3	60	0.00	16.66	83.3
3	350	1/3	60	0.00	13.81	86.2
4	375	1/3	60	0.00	10.17	89.8
5	400	1/3	60	0.00	9.56	90.4
6	375	1/3	30	0.00	10.28	89.7
7	375	1/3	90	0.00	9.80	90.2
8	375	1/1	60	0.00	24.25	75.8
9	375	1/2	60	0.00	16.72	83.3
10	375	1/3	60	1	9.89	90.1
11	375	1/3	60	3	9.63	90.4
12	375	1/3	60	6	9.33	90.7

*daf*, dry ash-free

When Table 4 was examined, as the solid/solvent ratio increased, the total conversion increased from 75.8% at 1/1 to 89.8% at 1/3, because as the amount of solvent increased, there was more hydrogen transfer to the biomass (Çavuşoğlu et al. 2022).

It was aimed in the liquefaction process to increase the amount of liquid product and reduce the amount of char. As seen in Table 4, the char efficiency which was 19.08% at 300 °C, decreased to 9.56% at 400 °C as expected. Similarly, char yield decreased from 10.28% at 30 min reaction time, to 9.80% at 90 min. In the solid/solvent ratio, the char efficiency decreased from 24.25% at 1/1 to 10.17% at 1/3. When the effect of catalyst concentration was examined, it was observed that the char efficiency decreased slightly as the catalyst concentration increased. However, in the experiments carried out under the same conditions, the char efficiency, which was 10.17% for the experiment without a catalyst, decreased by 9.33% when a 6% catalyst was used.

**Fig. 12:**
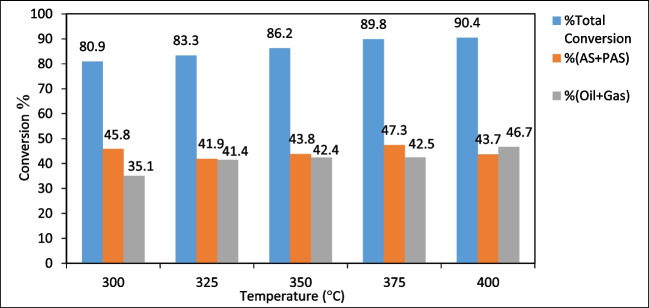
The effect of reaction temperature on conversion (reaction time: 60 min, initial nitrogen pressure: 20 bar, solid/liquid ratio: 1/3)

The effect of different parameters on (oil + gas) % and (PAS + AS) % was investigated in liquefaction experiments. When the effect of reaction temperature was examined, it was observed that (oil + gas) % increased and (PAS + AS) % decreased with increasing temperature (Fig. 12). For this reason, the optimum temperature was found to be 400 °C considering the total conversion and (oil + gas) % conversion (Ersöz 2023).

**Fig. 13:**
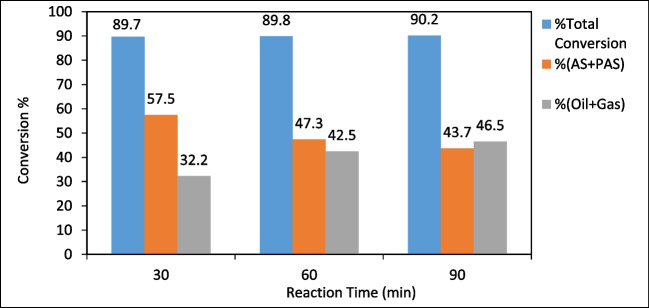
The effect of reaction time on conversion (temperature: 375ºC, initial nitrogen pressure: 20 bar, solid/liquid ratio: 1/3)

When the effect of reaction time was examined; it was observed that (oil + gas)% conversion increased and the amount of (PAS + AS)% decreased proportionally with the increase in reaction time. Since the total conversion remained relatively stable, it can be said that most of the (PAS + AS) % converted to (oil + gas) % (Coşkun 2020). Consequently, considering both total conversion and (oil + gas) %, the most suitable reaction time can be identified as 60 min (Fig. 13).

**Fig. 14:**
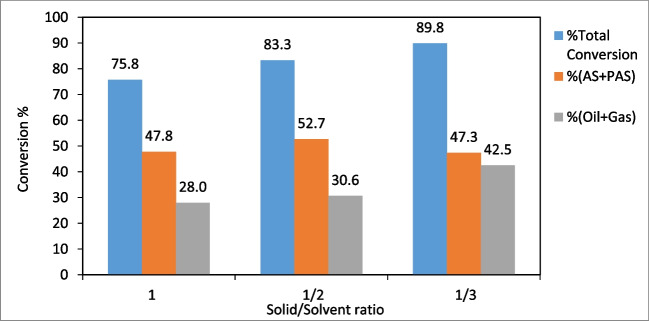
The effect of solid/solvent ratio on conversion (temperature: 375ºC, reaction time: 60 min, initial nitrogen pressure: 20 bar)

The effect of the solid/solvent ratio on total conversion, (oil + gas) % and (PAS + AS) % was shown in Fig. 14. As a result of the change in solid/solvent ratio from 1/1 to 1/3, there was a significant increase in total conversion and (oil + gas) %, while there was a decrease in (PAS + AS) %. However, as the amount of solvent increased, the amount of hydrogen transferred to the reaction medium increased significantly, resulting in a significant increase in the (oil + gas) %. Therefore, the most appropriate solid/solvent ratio can be considered as 1/3.

**Fig. 15:**
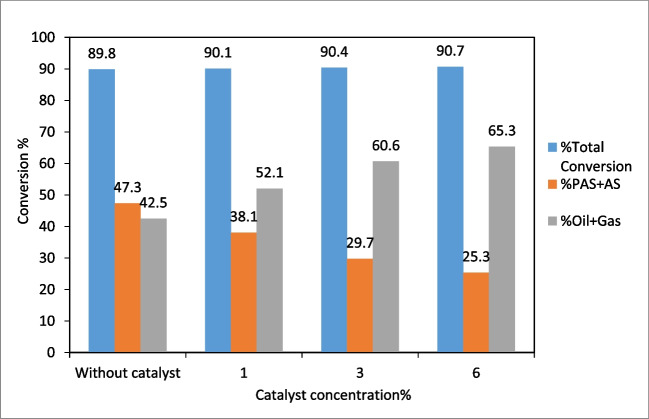
The effect of catalyst concentration on conversion (temperature: 375ºC, reaction time: 60 min, initial nitrogen pressure: 20 bar, solid/liquid ratio: 1/3)

When the effect of catalyst concentration on conversion was examined, it was seen that the (oil + gas) % ratio increased significantly with increasing catalyst concentration (Fig. 15). On the other hand, the total conversion remained almost unchanged and the amount of (PAS + AS) % has decreased very sharply. This result showed that as (PAS + AS) % decreased, (oil + gas) % formation increased. It was seen that the difference between %(PAS + AS) % and (oil + gas) % became larger with increasing catalyst concentration in Fig. 15. Therefore, the optimum catalyst concentration can be taken as 6% considering the total conversion and (oil + gas) % formation.

### Analysis of the biochars

Elemental analysis and calorific values of chars, the solid products obtained as a result of liquefaction, were given in Table 5. While the C, H, N, and S values of chars decreased compared to those of WS, the oxygen content increased.

**Table 5:** Elemental analysis and calorific values of chars

Temp./solid:solvent/time/catalyst conc	C%	H%	N%	S%	O*%	(H/C)_atomic_	Higher heating value(cal/g)
300 °C/1:3/60	64.98	5.08	0.84	0.07	29.03	0.94	5979
325 °C/1:3/60	65.15	5.16	0.87	0.11	28.71	0.95	5786
350 °C/1:3/60	55.91	4.68	0.66	0.12	38.63	1.00	6130
375 °C/1:3/60	60.08	4.31	0.60	0.05	34.95	0.86	6033
400 °C/1:3/60	53.12	4.28	0.67	0	41.93	0,97	6029
375 °C /1:3/30	63.19	3.98	0.51	0.05	32.27	0.76	6081
375 °C/1:3/60	60.08	4.31	0.60	0.05	34.95	0.86	6033
375 °C /1:3/90	54.74	3.84	0.49	0.04	40.90	0.84	6021
375 °C /1:1/60	49.71	3.91	0.46	0.05	45.87	0.94	7003
375 °C /1:2/60	67.95	4.09	0.52	0.08	27.35	0.72	6554
375 °C/1:3/60	60.08	4.31	0.60	0.05	34.95	0.86	6033
375 °C/1:3/60/%1	47.72	3.76	0.31	0.11	48.10	0.94	5633
375 °C/1:3/60/%0	60.08	4.31	0.60	0.05	34.95	0.86	6033
375 °C/1:3/60/%3	56.89	3.35	0.53	0	39.16	0.71	5287
375 °C/1:3/60/%6	45.41	2.91	0.41	0.04	51.23	0.77	4250

The increase in the amount of oxygen in the char showed that the solid product was enriched in minerals found in the WS (Karaca and Ceylan 2002). As observed in Table 5, the C, H, and N content of the solid products increased with decreasing solid/solvent ratio, temperature, and reaction time values. This increase was due to the radicals obtained by the fragmentation of molecules in the liquefaction process reassembling into high molecular weight products without being saturated with hydrogen. The fuel value of the chars formed by catalytic liquefaction is lower than that of non-catalytic liquefaction. This is because catalytic liquefaction results in higher conversion rates and lighter liquid products (Karaca et al. 2001; Karaca and Ceylan 2002; Karaca 2005).

When the elemental analysis results were examined, the amounts of carbon and hydrogen were generally high at low temperatures. While the conversion to liquid products increased as the temperature and reaction time increased, the carbon and hydrogen ratio of the chars decreased. Similarly, liquefaction efficiency increased under catalytic conditions where total conversion was high. Therefore, the amounts of carbon and hydrogen were lower than in non-catalytic conditions (Olam and Karaca 2023). While the (H/C)_atomic_ ratio of less than 1 indicated that the chars were compatible with solid fuels, 1 < (H/C)_atomic_ < 2 showed that they have similar thermal properties with liquid fuels. According to these results, chars were more similar to solid fuels in terms of their thermal properties (Önal 2015).

The upper heating value of WS used as biomass was 3646 cal/g. When the effect of temperature and reaction time under non-catalytic conditions was analyzed, the upper heating values were around 6000 cal/g, which was very high compared to the initial WS. Similarly, the upper heating values varied between 6000 and 7000 cal/g in experiments showing the effect of the solid/solvent ratio. As the amount of solvent increased, the total conversion amounts increased and the upper calorific values decreased. In the experiments under catalytic conditions, the upper heating values were between 4200 and 5600 cal/g and it was observed that the upper heating values decreased as the catalyst ratio increased. It was an expected result that the upper heating values decreased as % total conversion and (oil + gas) % efficiency increased. According to the results, biochars can be used as fuel or re-evaluated by liquefaction and gasification methods.

### Analysis of the bio-oil products

The contents of bio-oils obtained by liquefaction of WS under catalytic and non-catalytic conditions were characterized by GC/MS. As shown in Table 6, 7, 8, and 9, the bio-oil was mainly composed of aromatic compounds (benzene, indene and their derivatives) and polyaromatic compounds (naphthalene and derivatives). 1,2,3,4-Tetrahydro naphthalene (Tetralin), the most predominant in the tables, was used as solvent in liquefaction. The part that could not be removed by distillation remained in the bio-oil. Azulene, the second most common compound in the tables, is an aromatic hydrocarbon. It was observed that its amount in the bio-oil increased with the use of a catalyst. Azulene was used in optoelectronic devices with its physicochemical properties and in medicine with its anti-inflammatory properties (Bakun et al. 2021). Other substances found in high amounts in terms of % quality and % area in bio-oils were indane and its derivatives, benzene, butyl-, naphthalene, decahydro-, cis-, naphthalene, 1-methyl-. Among these compounds, it was found that “naphthalene, decahydro-, cis-” was among the compounds belonging to diesel, while the others were seen to be more compatible with gasoline compounds (Coşkun 2020).

**Table 6:** GC/MS analysis of the bio-oil obtained under non-catalytic conditions (375 °C: 60 min: 1/3 solid/solvent)

Peak no	Retention time (min)	Area (%)	Possible compounds	Quality (%)
1	11.13	0.03	Hydroperoxide, 1-ethyl butyl	90%
2	11.49	0.04	Hydroperoxide, 1-methyl pentyl	88%
3	12.47	0.03	Phenyl alcohol	92%
4	13.05	0.03	Decane < n- >	94%
5	15.11	0.16	Naphthalene, decahydro-	96%
6	15.18	0.05	Benzene, butyl-	91%
7	16.34	0.31	Indan, 1-methyl-	96%
8	16.46	0.04	Guaiacol	95%
9	16.79	0.38	Naphthalene < cis-decahydro- >	94%
10	17.90	0.11	Bicyclo[4.3.0]nonane, 2-methylene-, cis-	93%
11	19.26	87.79	Naphthalene, 1,2,3,4-tetrahydro-	89%
12	19.96	10.16	Azulene	98%
13	20.30	0.04	2-Methoxy-4-methyl phenol	84%
14	20.69	0.07	Mesityl alcohol	96%
15	21.10	0.04	Naphthalene, 1,2,3,4-tetrahydro-2-methyl-	89%
16	21.43	0.14	Naphthalene, 1,2,3,4-tetrahydro-1-methyl-	93%
17	21.73	0.08	2-Ethyl-2,3-dihydro-1H-indene	84%
18	22.32	0.06	1H-Indene, 2,3-dihydro-4,7-dimethyl-	96%
19	23.34	0.04	Phenol, 4-ethyl-2-methoxy-	90%
20	24.43	0.14	Naphthalene, 2-methyl-	96%
21	24.86	0.05	Naphthalene, 1-ethyl-1,2,3,4-tetrahydro-	96%
22	51.74	0.08	1-N-Pentyl-1,2,3,4-tetrahydronaphthalene	86%
23	51.87	0.08	Naphthalene, 1,2,3,4-tetrahydro-1-octyl-	84%
24	53.28	0.06	2,2'-Binaphthalene,1,1',2,2',3,3',4,4'-octahydro	92%

**Table 7:** GC/MS analysis of the bio-oil obtained under catalytic conditions (375 °C: 60 min: 1/3 solid/solvent: 1% of catalyst concentration

Peak no	Retention time (min)	%	Area (%)	Possible compounds	Quality (%)
1	5.21		0.02	Toluene	96%
2	11.13		0.04	Hydroperoxide, 1-ethyl butyl	92%
3	11.49		0.05	Hydroperoxide, 1-methyl pentyl	91%
4	12.47		0.04	Phenyl alcohol	93%
5	13.05		0.04	Dodecane	94%
6	14.38		0.05	1H-Indene, 2,3-dihydro-	93%
7	14.49		0.03	2-Cyclopenten-1-one, 2,3-dimethyl-	87%
8	15.11		0.16	Naphthalene, decahydro- >	96%
9	15.19		0.18	Benzene, butyl-	96%
10	16.34		1.07	1H-Indene, 2,3-dihydro-1-methyl -	96%
11	16.79		0.34	Naphthalene, decahydro-, cis-	94%
12	17.90		0.09	Bicyclo[4.3.0]nonane, 2-methylene-, cis-	92%
13	19.25		82.24	Naphthalene, 1,2,3,4-tetrahydro-	90%
14	19.97		14.82	Azulene	98%
15	21.43		0.16	Naphthalene, 1,2,3,4-tetrahydro-1-methyl-	94%
16	23.85		0.11	Naphthalene, 2-methyl-	91%
17	24.43		0.23	Naphthalene, 2-methyl-	97%
18	24.86		0.10	Naphthalene, 1-ethyl-1,2,3,4-tetrahydro-	95%
19	27.29		0.05	Naphthalene, 2-ethyl-	94%
20	27.96		0.06	Naphthalene, 1,2,3,4-tetrahydro-1-propyl-	92%
21	51.74		0.03	Naphthalene, 1,2,3,4-tetrahydro-1-propyl-	84%
22	51.87		0.04	Benzene, (1,2-di-cyclopropyl-2-phenyl ethyl)-	79%
23	53.28		0.08	2,2'-Binaphthalene, 1,1',2,2',3,3',4,4'-octahydro-	91%

**Table 8:** GC/MS analysis of the bio-oil obtained under catalytic conditions (375 °C: 60 min: 1/3 solid/solvent: 3% of catalyst concentration

Peak no	Retention time (min)	Area (%)	Possible compounds	Quality (%)
1	5.21	0.02	Toluene	97%
2	5.80	0.03	Cyclopentanone	88%
3	7.29	0.03	Cyclopentanone, 2-methyl-	95%
4	7.49	0.01	Cyclopentanone, 3-methyl-	88%
5	12.47	0.06	Phenyl alcohol	94%
6	13.05	0.04	Decane	95%
7	15.10	0.18	Naphthalene, decahydro-	97%
8	15.18	0.17	Benzene, butyl-	96%
9	15.99	0.07	p-Cresol	97%
10	16.34	0.91	Indan, 1-methyl-	96%
11	16.79	0.39	Naphthalene, decahydro-, cis-	93%
12	17.90	0.11	Bicyclo[4.3.0]nonane, 2-methylene-, cis-	93%
13	19.28	83.21	Naphthalene, 1,4,5,8-tetrahydro-	88%
14	19.98	13.61	Azulene	96%
15	20.69	0.07	Mesityl alcohol	97%
16	21.10	0.05	Naphthalene, 1,2,3,4-tetrahydro-2-methyl-	91%
17	21.43	0.16	Naphthalene, 1,2,3,4-tetrahydro-1-methyl-	93%
18	21.73	0.08	2-Ethyl-2,3-dihydro-1H-indene	85%
19	22.31	0.06	1H-Indene, 2,3-dihydro-4,7-dimethyl-	95%
20	23.84	0.12	Naphthalene, 2-methyl-	92%
21	24.01	0.04	Tridecane	96%
22	24.43	0.22	Naphthalene, 2-methyl-	97%
23	24.86	0.09	Naphthalene, 1-ethyl-1,2,3,4-tetrahydro-	96%
24	27.29	0.05	Naphthalene, 2-ethyl-	95%
25	27.96	0.05	Naphthalene, 1,2,3,4-tetrahydro-1-propyl-	93%
26	51.74	0.05	Naphthalene, 1,2,3,4-tetrahydro-1-propyl-	84%
27	51.87	0.05	Naphthalene, 1,2,3,4-tetrahydro-1-propyl-	82%
28	53.28	0.09	2,2'-Binaphthalene, 1,1',2,2',3,3',4,4'-octahydro-	92%

**Table 9:** GC/MS analysis of the bio-oil obtained under catalytic conditions (375 °C: 60 min: 1/3 solid/solvent: 6% of catalyst concentration

Peak no	Retention time (min)	Area (%)	Possible compounds	Quality (%)
1	5.21	0.02	Toluene	93%
2	5.80	0.02	Isopropyl 4-pentanoate	81%
3	7.29	0.03	Cyclopentanone, 2-methyl-	95%
4	7.49	0.01	Cyclopentanone, 3-methyl-	85%
5	9.07	0.01	1,10-Dichlorodecane	74%
6	9.31	0.01	Nonane	85%
7	9.54	0.01	Cyclohexane, 1-(1,1-dimethyl ethyl)-4-methyl-	71%
8	11.13	0.03	Hydroperoxide, 1-ethyl butyl	91%
9	11.49	0.04	Hydroperoxide, 1-methyl pentyl	92%
10	12.47	0.05	Phenyl alcohol	94%
11	13.05	0.04	Decane	94%
12	14.38	0.04	Indane	95%
13	15.11	0.16	Naphthalene, decahydro-	96%
14	15.18	0.18	Benzene, butyl-	96%
15	15.99	0.06	p-Cresol	95%
16	16.33	1.05	Indan, 1-methyl-	97%
17	16.79	0.35	Naphthalene < cis-decahydro- >	94%
18	17.90	0.09	Tricyclo[4.4.0.0(2,8)]decane	93%
19	19.25	83.07	Naphthalene, 1,2,3,4-tetrahydro-	89%
20	19.97	13.70	Azulene	98%
21	20.69	0.07	Phenol, 2,3,6-trimethyl-	96%
22	21.10	0.05	Naphthalene, 1,2,3,4-tetrahydro-2-methyl-	89%
23	21.43	0.15	Naphthalene, 1,2,3,4-tetrahydro-1-methyl-	94%
24	21.73	0.08	2-Ethyl-2,3-dihydro-1H-indene	83%
25	22.32	0.06	1H-Indene, 2,3-dihydro-4,7-dimethyl-	96%
26	23.85	0.08	Naphthalene, 2-methyl-	92%
27	24.01	0.03	Tridecane	96%
28	24.43	0.20	Naphthalene, 2-methyl-	97%
29	24.86	0.08	Naphthalene, 1-ethyl-1,2,3,4-tetrahydro-	96%
30	27.28	0.04	Naphthalene, 2-ethyl-	94%
31	27.96	0.05	Naphthalene, 1,2,3,4-tetrahydro-1-propyl-	94%
32	51.74	0.03	Benzene, (1,2-di-cyclopropyl-2-phenyl ethyl)-	82%

Table 10 displayed the GC/MS results of the liquefaction products of various biomass types, gasoline, diesel, and WS in the literature. According to this table, it was found that the chemical and light product content of the obtained liquid fuel products is more valuable compared to the results in the literature. When compared with the GC–MS results of gasoline and diesel, it was observed that the obtained liquid products contained a few light components found in gasoline but were closer in content to diesel. This situation indicated that a product consisting of light compound components was obtained between gasoline and diesel (Olam and Karaca 2022). Considering the conversion rate, fuel properties, and the production of valuable chemicals, it was thought that this process would be feasible/applicable. According to the IEA Bioenergy 2023 report, 11 commercial and 15 demonstration-scale plants are in operation or under construction/development in 14 countries. After the commissioning of the first commercial plant in 2023, most of the obtained liquid products have been utilized for heating purposes, while efforts are underway to explore other potential applications (Collard et al. 2023). By improving parameters such as catalysts, hydrogen sources, etc., it is possible to increase the content of light products and enhance the production of high-quality fuel and valuable chemicals.

**Table 10:** Effectiveness of liquid product

Biomass type	Possible products in GC–MS analysis		Reference
Chlorella algal	• Indane • Decane • Tridecane • Phenol	• Naphthalene • 2-Methyl naphthalene • 1,2,3,4-Tetrahydro naphthalene • 4-Ethylphenol	**(**Rathsack et al. 2019**)**
Cellulose	• Phenol • p-cresol • 2,3,4,5,6,7-hexahydro-1H-inden-1-one	• Ethyl phenol • Indane • 2-methyl-1-indan	**(**Villadsen et al. 2012**)**
Waste paper	• Naphthalene • Naphthalene, 1-ethyl-naphthalene	• 1H-Indene,2,3-dihydro-4,7-dimethyl- • 1-ethyl-2,3-dihydro-1-methyl-1H-indene	**(**Karaca and Koyunoğlu 2010**)**
Pinewood sawdust	• 2-methyl-naphthalene	• Phenol • 2-methoxy-4-methyl-phenol	**(**Wang et al. 2013**)**
Sunflower shell	• Toluene • Decane	• 3-ethyl-5-methyl-phenol	**(**Guo et al. 2008**)**
Paulownia	• Phenol • Phenol, 2-methoxy-4-methyl-	• Naphthalene, 2,3-dimethoxy- • Phenol, 4-methyl- • Benzene, 1,2,3-trimethoxy-5-methyl-	**(**Sun et al. 2010**)**
Palm empty fruit bunch	• Phenol • Naphthalene • Decahydro naphthalene	• 1-(2-methylprop-1-enyl)benzene	**(**Koriakin et al. 2017**)**
Peach seed kernel	• Benzene. 1-Ethenyl-3-Ethyl • Phenol	• 1 H-Indene 2,3-Dihydro-4- Propyl • Naphthalene 1,2-Dihydro	**(**Gündüz et al. 2023**)**
Gasoline	• Toluene • 1,3-dimethyl-Benzene • p-Xylene	• 1-methyl-3-propyl-Benzene • 1,2,3-trimethyl-Benzene •1-ethyl-2-methyl-Benzene	**(**Olam and Karaca 2022**)**
Diesel	• N-Decane • Dodecane • Tridecane • Tetradecane • Naphthalene, 1,2,3,4-Tetrahydro-1,4-Dimethyl-	•Benzene, butyl-, •Naphthalene, decahydro-, cis-, •Naphthalene, 1-methyl- 2 h-Inden-2-One, 1,3-•Dihydro-1,1,3,3-Tetramethyl-	**(**Olam and Karaca 2022**)**
Walnut shells	• Toluene • Decane • p-Cresol • Phenol • Tridecane • Azulene • Phenol, 2-methoxy-4methyl- • Dodecane	• Naphthalene, 1,2,3,4-tetrahydro-2-methyl- • Naphthalene, 1,2,3,4-tetrahydro-1-methyl- • 2-Ethyl-2,3-dihydro-1H-indene • 1H-Indene, 2,3-dihydro-4,7-dimethyl- • Naphthalene, 2-methyl-	**In this study**

**Fig. 16:**
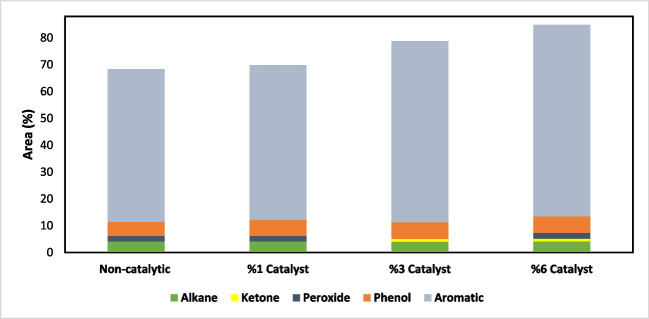
Selectivity of light liquid product

The distribution of the main components in the bio-oil obtained by liquefying WS for different catalyst concentrations is illustrated in Fig. 16. The main components of the bio-oils were found to be aromatics, ketones, phenolics, alkanes, and peroxides (Yang et al. 2014). As the catalyst concentration increased, the content of hydrocarbons increased due to depolymerisation of the macromolecular compound. On the other hand, there was a decrease in compounds with high carbon numbers, while the number of compounds of various types increased (Zhao et al. 2023).

The catalyst facilitates the hydrolysis and fragmentation of biomass components, leading to the formation of phenolic compounds by breaking the ether bonds present in its structure. The catalyst prevents the recombination of biomass that has been broken down into radicals by increasing the activity of hydrogen (Zhou et al. 2016; Kumar et al. 2023). As can be seen from Table 7, 8, 9, and 10, the composition of bio-oils was significantly influenced by the solvent used. It has been observed that the use of tetralin increases the variety of chemical species in bio-oil and enriches it aromatically (Jitpinit et al. 2024).

## Conclusions

In this study, the synthesis of SF/NZVI catalyst and its efficiency in the liquefaction of WS were investigated. The results were given as follows:

- The SF/NZVI catalyst synthesized by liquid phase reduction was characterized by SEM–EDX, TEM, BET, FTIR, XPS, and XRD. Analysis revealed nano iron structures bonded to the SF surface.
- The effect of different parameters on the liquefaction process of WS was investigated. (Oil + gas) % yield increased from 35.1 to 46.7% when the temperature was raised from 300 to 400 °C, from 32.2 to 46.5% when the reaction time was increased from 30 to 90 min, and from 28.0 to 47.5% when the solid/solvent ratio was changed from 1/1 to 1/3. In liquefaction under catalytic conditions, the (oil + gas) % increased from 52.1 to 65.3% as catalyst concentration ranged from 1 to 6%, and it was obtained 42.5% for non-catalytic conditions under the same liquefaction conditions According to these results, the optimum conditions for liquefaction of WS can be taken as 400 °C temperature, 60 min reaction time, 1/3 solid/solvent ratio, and catalyst concentration of 6%. GC–MS analyses showed that bio-oils, which are liquefaction products, were composed of compounds with significant fuel properties. According to quality % and area %, the results were found to be closer to gasoline compared to diesel.
- The experimental results showed that the SF/NZVI catalyst was highly efficient in the direct liquefaction of WS and that the obtained bio-oils could be used as an alternative fuel or as a raw material in the chemical industry.

## Data Availability

The data will be made available on request from the corresponding author.
